# Unbalanced circulating Humanin levels and cardiovascular risk in chronic hemodialysis patients: a pilot, prospective study

**DOI:** 10.1007/s40620-024-02032-4

**Published:** 2024-08-05

**Authors:** Davide Bolignano, Marta Greco, Pierangela Presta, Anila Duni, Mariateresa Zicarelli, Simone Mercuri, Efthymios Pappas, Lampros Lakkas, Michela Musolino, Katerina K. Naka, Sara Pugliese, Roberta Misiti, Daniela Patrizia Foti, Michele Andreucci, Giuseppe Coppolino, Evangelia Dounousi

**Affiliations:** 1 Nephrology and Dialysis Unit, Magna Graecia University, Catanzaro, Italy; 2 Department of Medical and Surgical Sciences-Renal Unit, University “Magna Graecia”, Campus Salvatore Venuta, Viale Europa, 88100 Catanzaro, Italy; 3 Department of Health Sciences, Magna Graecia University, Catanzaro, Italy; 4 Clinical Pathology Lab, Magna Graecia University, Catanzaro, Italy; 5 Department of Nephrology, School of Medicine, University of Ioannina, Ioannina, Greece; 6 Hemodialysis Unit, General Hospital of Filiates, Filiates, Greece; 7 Physiology Department, Faculty of Medicine, School of Health Sciences, University of Ioannina, Ioannina, Greece; 8 Second Department of Cardiology, University Hospital of Ioannina, Ioannina, Greece; 9 Experimental and Clinical Medicine, Magna Graecia University, Catanzaro, Italy

**Keywords:** End-stage kidney disease, Hemodialysis, Cardiovascular risk, Biomarker, Humanin

## Abstract

**Background:**

Mortality and cardiovascular (CV) risk prediction in individuals with end-stage kidney disease (ESKD) on chronic hemodialysis (HD) remains challenging due to the multitude of implicated factors. In a multicenter ESKD-HD cohort, we tested the prognostic yield of the assessment of circulating Humanin, a small mitochondrial-derived peptide involved in CV protection, on CV events and mortality.

**Methods:**

We conducted a prospective, observational, pilot study on 94 prevalent HD patients. The prognostic capacity of circulating Humanin levels was tested on a primary composite (all-cause mortality + non-fatal CV events) and a secondary exploratory endpoint (all-cause mortality alone).

**Results:**

Baseline Humanin level was comparable in patients reaching the primary or secondary endpoint as compared to others (*p* = 0.69 and 0.76, respectively). Unadjusted followed by multivariable Cox regression analyses adjusted for age, left ventricular mass index (LVMi), *E*/*e*’, pulse pressure and diabetes mellitus indicated a non-linear relationship between Humanin levels and the composite outcome with the highest Hazard Ratio (HR) associated with very low (< 450.7 pg/mL; HR ranging from 4.25 to 2.49) and very high (> 759.5 pg/mL; HR ranging from 5.84 to 4.50) Humanin values. Restricted cubic splines fitting univariate and multivariate Cox regression analyses visually confirmed a curvilinear trend with an increasing risk observed for lower and higher Humanin values around the median, respectively. A similar, u-shaped association was also evidenced with the secondary endpoint.

**Conclusions:**

Altered Humanin levels may impart prognostic information in ESKD-HD patients at risk of death or CV events. Future investigations are needed to confirm whether Humanin measurement could improve CV and mortality risk prediction beyond traditional risk models.

**Graphical abstract:**

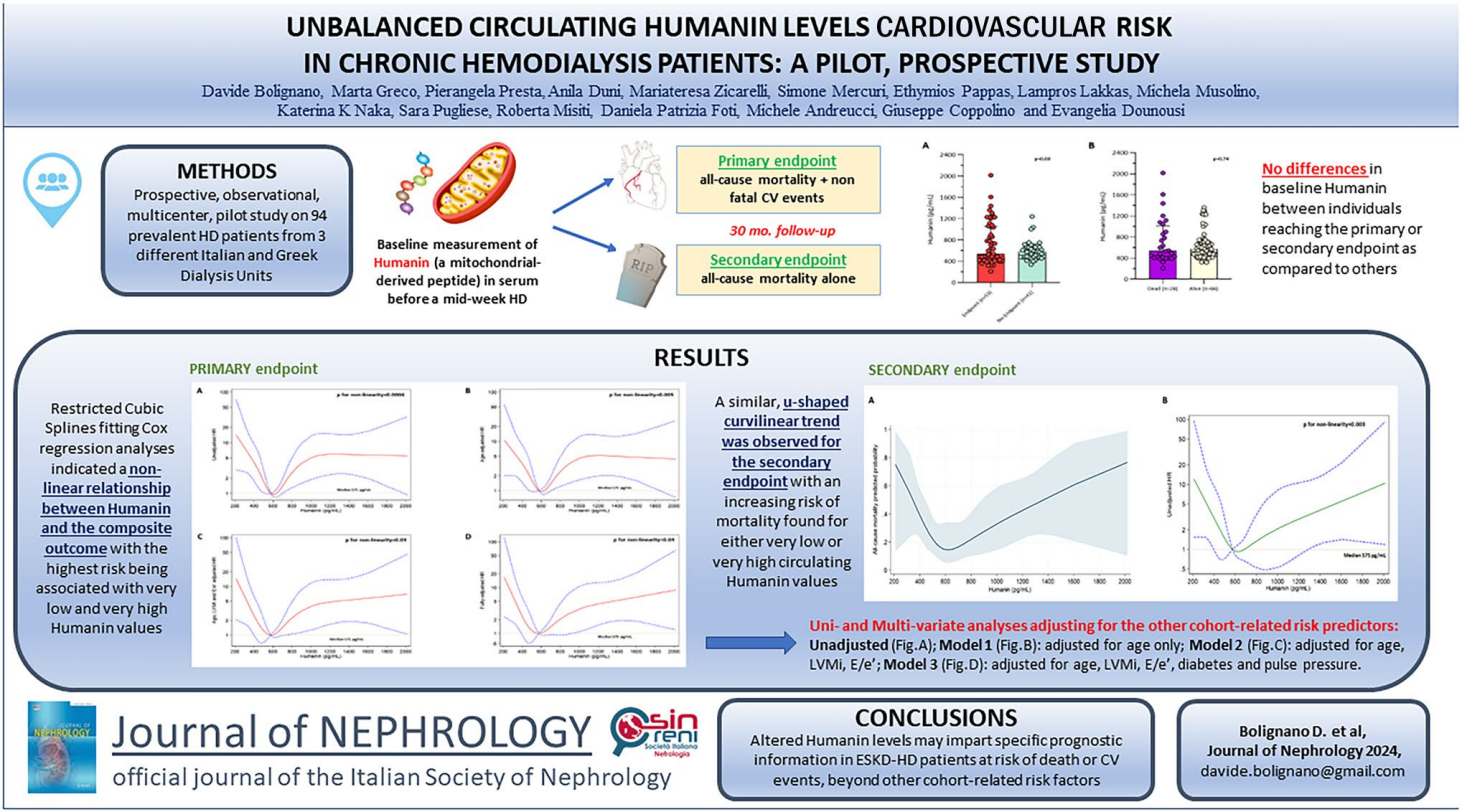

**Supplementary Information:**

The online version contains supplementary material available at 10.1007/s40620-024-02032-4.

## Introduction

People with end-stage kidney disease (ESKD) receiving chronic hemodialysis (HD) are remarkably predisposed to cardiovascular (CV) complications, but risk prediction in this setting remains challenging due to the complexity of the factors involved [1].

Humanin, a small bioactive peptide encoded by the mitochondrial genome, has recently garnered significant attention as a potent antioxidant and anti-apoptotic agent involved in the regulation of metabolic and energy homeostasis [2].

Humanin is found in the bloodstream, mostly released by skeletal and cardiac muscle cells, astrocytes, endothelial cells and gonads following mitochondrial activation [3] and its deregulation has been implicated in the pathogenesis of various neurodegenerative and lung diseases, diabetes mellitus and even of cancer [4].

In experimental models, Humanin exerts neuroprotective effects [5], enhances longevity [6, 7], and may attenuate CV damage by reducing myocardial ischemia–reperfusion injury, improving endothelial function and delaying myocardial senescence and atherosclerosis progression [8].

In clinical studies, low plasma Humanin values parallel the severity of coronary endothelial dysfunction [9] and can predict major adverse cardiac events in individuals with ischemic cardiac disease, thus representing a candidate biomarker for anticipating worse CV outcomes [10].

Although altered Humanin plasma levels have been observed in pre-dialysis ESKD patients [11], to date, this substance has never been investigated in the more complex ESKD-HD setting, particularly in its relationship with the exceeding CV risk that characterizes this condition.

Starting from these premises, we conducted a pilot study in a multicenter cohort of prevalent HD patients to evaluate the clinical significance and prognostic value of circulating Humanin levels regarding the risk of mortality and CV events, on top of other cohort-related risk factors.

## Methods

### Study criteria

We designed a pilot, prospective, observational, multicenter cohort study of chronic HD patients undergoing a regular thrice-weekly treatment regimen at the “Renato Dulbecco” University Hospital of Catanzaro, Italy, the University Hospital of Ioannina and the General Hospital of Filiates, Greece.

Inclusion criteria for enrollment were dialysis vintage > 6 months, stable dry-weight with a normotensive edema-free state, and an unmodified therapeutic scheme over the 3 months before the start of the study. The main exclusion criteria were acute or non-intermittent hemodialysis, a recent switch from peritoneal dialysis or kidney transplantation (< 12 months), recent hospitalization (< 1 month) for CV disease, severe obesity (BMI > 40) or malnutrition (BMI < 15), active neoplasia, liver or infectious diseases, alterations in leukocyte count and/or treatment with steroids or immunosuppressors.

### Baseline assessment

Anthropometric, clinical, biochemical, and dialysis data were collected before a mid-week dialysis session. Blood pressure values were measured three times consecutively before the dialysis session started by an automated sphygmomanometer, and the average values were considered for data analysis. Additionally, a complete echocardiographic evaluation was performed by a skilled operator unaware of the patients’ characteristics. All laboratory parameters were measured following the standard methods used in the routine clinical laboratory. The natural logarithm of the ratio between initial and final urea concentration (KT/V) was calculated to assess dialysis adequacy.

Further blood samples were collected from a peripheral vein before starting dialysis and centrifuged at 1227 g for 15 min at 4 °C with the aliquots being stored at − 80 °C until thawed for batch analysis. Humanin was measured in the serum using a commercially available ELISA kit (My Biosource, San Diego, CA, USA), according to the manufacturer’s instructions. The enzymatic reactions were quantified in an automatic microplate photometer and were all performed in the same laboratory (Clinical Pathology Lab of the “Renato Dulbecco” University Hospital- Catanzaro, Italy). Measurements were made blind and in duplicate and Humanin levels were expressed as pg per mL.

### Longitudinal phase

The prospective phase consisted of a planned 30-month follow-up, starting after the baseline assessment. The primary study endpoint was a composite of all-cause mortality and non-fatal CV events including coronary, cerebrovascular and peripheral artery disease events, acute heart decompensation and severe cardiac arrhythmia episodes requiring hospitalization. Additionally, all-cause mortality alone was investigated as a secondary, exploratory endpoint. Event-free individuals who eventually received a kidney graft before the end of follow-up were censored at the date of kidney transplantation.

### Statistical analysis

All statistical analyses were performed using the STATA (version 18.0; StataCorp LL, Texas, US) and the GraphPad Prism (version 9.0.0; GraphPad Software LLC) software. Data were presented as mean and SD, median and interquartile range, and frequency distribution.

For the primary combined endpoint, longitudinal analyses were conducted on a time-to-first-event basis. Possible linear associations between Humanin levels and the study endpoints were first tested by standard Cox-proportional hazard regression analyses. To explore non-linear relationships, patients were stratified for quartiles of Humanin levels, considering the group with the lowest outcome incidence as the reference category. Crude and adjusted hazard ratios were computed by univariate followed by multivariate Cox regression analyses adjusting for other variables previously identified as independent predictors of the composite outcome. Possible multicollinearity was tested by the variance inflation factor (VIF), considering a cut-off of VIF > 4. Regression models were also adjusted by cohort type to account for possible differences in the background risk.

For the secondary, exploratory endpoint, crude linear associations between Humanin and all-cause mortality were assessed by univariate logistic and Cox regression analyses.

Restricted cubic splines (RCS) were elaborated with 3 knots placed at the 10th, 50th, and 90th percentiles, as recommended by Harrell [12] to visually explore the shape of non-linear associations between Humanin and the study endpoints.

For the primary endpoint, restricted cubic splines were fitted by unadjusted followed by multivariate Cox regression analyses, considering the median of Humanin levels as the reference value for the hazard ratio.

The fit of the spline models was tested against that of corresponding standard linear models by the likelihood-ratio test considering *p* values < 0.05 as indicative of a better capacity of the cubic spline model to describe the risk distribution. The Akaike information criterion (AIC) and the Bayesian information criterion (BIC) were computed to assess the quality of model fitting.

For the secondary endpoint, restricted cubic splines were fitted by crude logistic regression analyses to display the predicted probability of all-cause mortality and by unadjusted Cox regression to assess the non-linear, time-dependent risk variation across Humanin values. All statistical analyses were considered significant for *p* values < 0.05.

## Results

### Study population characteristics

The study cohort included 94 ESKD patients on chronic HD treatment. Among these, 30 underwent dialysis at the “Renato Dulbecco” University Hospital of Catanzaro (Italy), 37 at the University Hospital of Ioannina (Greece), and 27 at the General Hospital of Filiates (Greece). The Italian and Greek sub-cohorts did not display significant differences for comorbidities or with regard to the main clinical, laboratory, and instrumental parameters recorded.

The mean age of the whole study cohort was 68 ± 12.8 years and male gender was prevalent (63.9%). Twenty-six patients (27.6%) were diabetic and roughly half (*n* = 48) reported a previous history of at least one CV disorder. Fifty-four patients (57.4%) followed a standard bicarbonate dialysis regimen, while the remainder were treated by hemodiafiltration. The median dialysis vintage was 33 months (IQR 17–75.2) with satisfactory mean dialysis adequacy (mean kT/V: 1.42 ± 0.27). Echocardiography indicated, on average, the presence of diastolic dysfunction (*E*/*e*’ 10.8 [7.9–12.2]) and increased left ventricular mass values ([LVMi] 136.8 ± 38.8 g/m^2^). The remaining laboratory, clinical, and echocardiography parameters were all within the normal or recommended ranges of therapeutic control for uremic individuals. Supplementary Table 1 summarizes the main characteristics of the study population.

### Prospective endpoints

Over a median follow-up of 26.5 months (range 1–30), 53 subjects (56.4%) reached the primary endpoint. Of these, 10 subjects died due to a fatal CV event, 12 due to non-CV causes, while non-fatal CV events occurred in the remaining 31 subjects (11 acute coronary events, 3 cerebrovascular disease, 5 acute heart decompensation, 7 severe arrhythmia episodes and 5 peripheral artery disease).

At baseline, patients experiencing the endpoint were more frequently diabetic (39.6 vs. 12.2%; *p* = 0.003) and older (71.2 ± 11.9 vs. 63.8 ± 12.8 years; *p* = 0.005) and displayed lower HDL cholesterol (40.3 ± 10.2 vs 46.3 ± 10.1 mg/dL; *p* = 0.03), diastolic (69.2 ± 12.1 vs. 75.5 ± 10.8 mmHg; *p* = 0.03) and pulse pressure levels (72.3 ± 23.7 vs. 65.1 ± 18.1 mmHg; *p* = 0.04), as well as worse left atrial volume (34.5[24.7–42.7] vs. 23.2[17.5–42.9]; mL/m^2^; *p* = 0.04), left ventricular mass index (141.9 ± 40.1 vs. 124.7 ± 38 g/m^2^; *p* = 0.03), diastolic dysfunction degree (*E*/*e*’: 11.9 [7.9–14.4] vs. 8.4 [6.2–11.6]; *p* = 0.03) and ejection fraction (55.9 ± 9.9 vs 59.6 ± 7.6%; *p* = 0.05). Among these clinical variables, exploratory multivariate logistic analyses (not shown) identified age, LVMi, *E*/*e*’, diabetes mellitus, and pulse pressure as significant independent predictors of the primary outcome in this study cohort.

Supplementary Table 1 summarizes clinical data in HD patients reaching the primary endpoint as compared to others.

For the secondary, exploratory endpoint (all-cause mortality), we recorded 6 more fatal events (4 due to CV and 2 due to non-CV causes; a total of 28/94 subjects; 29.8%) beyond those collected in the time-to-first-event analysis. The median follow-up duration for the secondary endpoint was 28 months (range 1–30).

### Humanin levels and study endpoints

No differences in median baseline Humanin levels were reported in patients reaching the primary endpoint as compared to others (543[419.2–1050.5] vs. 580 [476.2–667] pg/mL, *p* = 0.69; Supplementary Fig. 1a). Likewise, levels were statistically similar between individuals who died (secondary exploratory endpoint) as compared to those who were alive at the end of the prospective phase (538 [410–973] vs. 580.5 [473–719], *p* = 0.76; Supplementary Fig. 1b).

Univariate Cox regression analyses indicated a barely significant capacity of Humanin to predict the primary endpoint in a linear manner (Hazard Ratio [HR] 1.000; 95% Confidence Interval [CI] 0.999–1.001 for each 1 pg/mL increase in circulating levels; *p* = 0.08).

Conversely, patient stratification for quartiles of Humanin revealed a low outcome incidence (6/23 patients; 26.1%) among those with Humanin levels of 575–759.5 pg/mL (3rd quartile). Assuming this subgroup as the reference risk category, an increased crude HR of the combined endpoint was observed in either HD patients showing very low (< 450.7 pg/mL; HR 4.25; 95% Confidence Interval [CI] 1.52–11.86) or very high Humanin levels (> 759.5 pg/mL; HR 5.84; 95%CI 1.65–20.64), while such a risk was apparently similar in individuals with intermediate values (2nd quartile: 450–575 pg/mL; 1.41; 95%CI 0.47–4.22). In multivariate analyses, we built three different models adjusting for the variables identified in this cohort as independent risk predictors of the combined endpoint (age, LVMi, *E*/*e*’, pulse pressure, diabetes mellitus). Model 1 was adjusted for age only; Model 2 was adjusted for age and parameters of cardiac morpho-dysfunction (LVMi and *E*/*e*’); Model 3 was fully adjusted for age, LVMi, *E*/*e*’, pulse pressure, and diabetes mellitus.

All multivariate models confirmed an increased HR of the endpoint in individuals with Humanin levels falling into the first (HR ranging from 3.55 to 2.49) and the fourth quartile (HR ranging from 5.30 to 4.50), while the HR remained comparable to the reference group in those with intermediate values (2nd quartile). Multicollinearity was excluded (variance inflation factor values ranging from 1.80 to 3.62). Table 1 summarizes univariate and multivariate Cox-regression hazard ratios of the composite study endpoint across quartiles of Humanin levels.

**Table 1:** Univariate and multivariate Cox-regression hazard ratios of the composite study endpoint across quartiles of circulating Humanin levels

Model	Humanin < 450.7 pg/mL (*n* = 25)	Humanin 450.7–575 pg/mL (*n* = 23)	Humanin 575–759.5 pg/mL (*n* = 23)	Humanin > 759.5 pg/mL (*n* = 23)	*p* of the model
Unadjusted	4.25 (1.52–11.86)	1.41 (0.47–4.22)	*Reference*	5.84 (1.65–20.64)	0.0001
Model 1	3.55 (1.24–10.18)	1.31 (0.43–3.96)		5.30 (1.32–18.61)	0.0001
Model 2	2.48 (1.19–7.76)	1.11 (0.35–3.32)		4.74 (1.09–18.54)	0.001
Model 3	2.49 (1.01–7.49)	1.10 (0.35–3.41)		4.50 (1.21–18.73)	0.004

Model 1: adjusting for age; Model 2: adjusting for age, LVMi and *E*/*e*’; Model 3: fully-adjusted model (Model 2 + pulse pressure and diabetes). 3rd quartile of Humanin levels (575–759.5 pg/mL) selected as reference risk category because displaying the lowest incidence of the composite endpoint

**Fig. 1:**
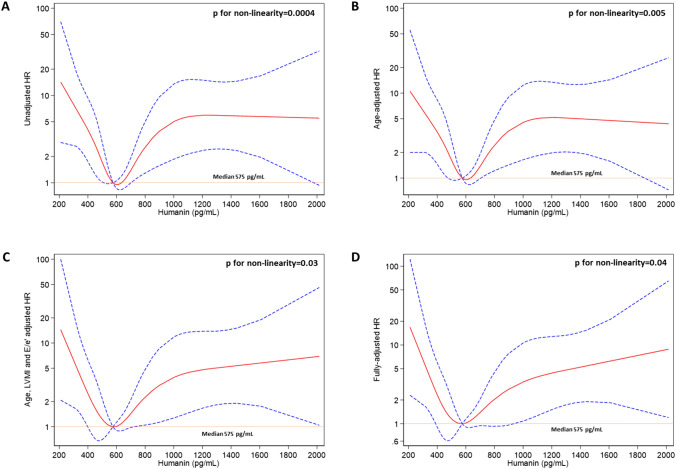
Restricted cubic splines (RCS) depicting the non-linear relationship between circulating Humanin levels and the primary endpoint. RCS were fitted by **A** univariate Cox regression analyses and multivariate Cox regression analyses adjusting for **B** age only; **C** age, LVMi and *E*/*e*’ values; **D** age, LVMi, *E*/*e*’, pulse pressure and diabetes (fully-adjusted model). Median Humanin levels (575 pg/mL) were considered as the reference risk value. The solid red line represents the point estimate for the HR and the dotted lines represent the 95% CIs

Restricted cubic splines of Humanin levels fitting Cox regression analyses for the primary endpoint (Fig. 1) described a peculiar curvilinear pattern of the estimated HR across Humanin levels (assuming the median value as the reference risk), with the highest risk probability observed in individuals displaying very low Humanin levels and an increasing risk evidenced in individuals with Humanin above the median, which remained constant at the highest values. Such a curvilinear shape was not affected by adjustment for age (multivariate model 1); conversely, the risk associated with Humanin levels above the median displayed an increasing tendency, rather than a stable pattern, in the model adjusting for age and the severity of cardiac morpho-dysfunction (model 2) as well as in the fully adjusted model. The likelihood-ratio test confirmed the capacity of all cubic spline models to describe the risk distribution better than the linear ones (*p* ranging from 0.0004 to 0.04; Table 2).

**Table 2:** Differences in model fitting between linear- and restricted cubic spline-fitted Cox-regression models testing the association between circulating Humanin levels and the composite study endpoint

Model	Linear fitting				RCS fitting				*p* for non-linearity
	*p*	*X*^*2*^	*AIC*	*BIC*	*p*	*X*^*2*^	*AIC*	*BIC*	
Unadjusted	0.08	3.09	445.1	447.6	0.0003	21.27	432.9	443.1	0.0004
Model 1	0.006	10.1	440.1	445.1	0.0003	23.13	433.1	445.8	0.005
Model 2	0.007	13.9	322.1	331.4	0.002	22.5	319.5	335.7	0.03
Model 3	0.02	15.1	324.9	338.4	0.005	23.5	322.5	343.4	0.04

Model 1: adjusting for age; Model 2: adjusting for age, LVMi and E/e’; Model 3: fully-adjusted model (Model 2 + pulse pressure and diabetes). AIC, Akaike information criterion; BIC, Bayesian information criterion

**Fig. 2:**
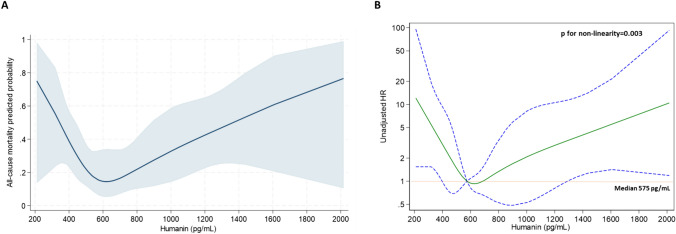
Restricted cubic splines of circulating Humanin levels **A** displaying the predicted probability of all-cause mortality from logistic regression (navy line with gray area showing the 95%CIs) and **B** fitting unadjusted Cox regression for mortality with median Humanin levels (575 pg/mL) considered as the reference risk value. The solid green line represents the point estimate for the HR and the dotted lines represent the 95% CIs

By the same token, in models fitting either logistic or Cox-regression analyses, a curvilinear, u-shaped association (*p* for non-linearity = 0.003) was described between Humanin levels and the secondary endpoint (all-cause mortality), with the strongest crude risk of death being observed, again, among individuals with either very low or very high baseline Humanin values (Fig. 2).

## Discussion

In this study, we found circulating Humanin as a novel potential biomarker for refining mortality and CV risk assessment in chronic HD patients.

Mitochondrial dysfunction is largely pervasive in ESKD-HD and contributes to CV disease exacerbation [13]; accordingly, low plasma levels of mitochondrial-derived peptides reflect the severity of such dysfunction and generally predict worse CV outcomes, in line with their protective role on the CV system [14].

Interestingly, in our study cohort, baseline Humanin levels were comparable between patients reaching the primary or the secondary endpoint as compared to others; this prevented the identification of single Humanin thresholds for risk discrimination and, in contrast with previous evidence, pointed to a non-linear relationship between this substance and the outcomes of interest.

Indeed, the prognostic association between Humanin values and the primary endpoint was poorly fitted by linear regression models; conversely, after patient categorization for quartiles of Humanin, the lowest frequency of endpoint occurrence was found among individuals falling within the intermediate quartile (575–759.5 pg/mL), while an increased risk was documented in those with either very low (< 450.7 pg/mL) or very high (> 759.5 pg/mL) values. By the same token, the visual exploration of this non-linear association by restricted cubic spline models evidenced a peculiar curvilinear trend with a steadily higher risk excess for Humanin levels above the median and a stronger, increasing risk associated with very low Humanin values. Importantly, this trend was not affected by age adjustment, thereby indicating that such a particular predictive pattern was independent of this potential confounder. This observation is, in our view, of foremost importance, given the strong modulating effect of age on both Humanin balance and CV events in the general population [6]. Conversely, more complex multivariate adjustments including the other cohort-related outcome predictors (namely- LVMi, *E*/*e*’, diabetes and pulse pressure) confirmed the strongest association of lower Humanin values with the endpoint but uncovered an increasing risk trend above the median, thus suggesting a synergic contribution of these factors on the overall risk estimation.

By the same token, a very similar curve was observed also at unadjusted risk analyses modeled on the secondary endpoint (all-cause mortality alone). This reinforces the unfavorable prognostic significance of displaying either very low or very high circulating Humanin in the specific ESKD-HD setting.

The observational nature of our investigation does not allow us to explain the biological meaning of these findings. In the general population, circulating Humanin decreases linearly with age [15], but paradoxically high values have been found in centenarians and their offspring [6]. Additionally, genetic variants of the Humanin gene have been associated with longevity in Ashkenazi ancestry [16], while the experimental overexpression of Humanin in a *Caenorhabditis elegans* model elicited a dramatic increase in its lifespan [17]. Hence, increased Humanin activity generally evokes a survival advantage over the normal aging processes and is thus assumed as a favorable find.

Nevertheless, although low circulating Humanin levels are usually considered detrimental and anticipate worse outcomes, high values, in specific conditions, may reflect an exaggerated mitochondrial response to a more sustained CV stress, eventually preluding to death, event occurrence, or disease exacerbation [18]. This hypothesis pairs well with the opinion that the effects of Humanin on health might largely be context-dependent [19] and supports the importance of finding measures to maintain an optimal systemic balance.

Our study has strengths and weaknesses that deserve acknowledgment. The main strengths are the multi-country cohort, a rigorous methodological approach, a prospective phase with adequate follow-up length and event rate to perform reliable multivariate analyses, and the establishment of solid and largely validated endpoints with a systematic event-collection. The main limitation of this study is represented by the limited sample size which prevented statistical overfitting but was insufficient to let us perform multivariate adjustments for the secondary endpoint. On top of this, the wide confidence intervals of HR at regression analyses may limit the certainty of the evidence, thus hampering the generalizability of our findings, while the observational nature of the study cannot prevent the presence of selection bias or residual confounding.

In conclusion, we found altered Humanin levels to impart potentially relevant prognostic information in ESKD-HD patients at risk of death or CV events. Future investigations on larger and more heterogeneous cohorts are needed to confirm whether circulating Humanin measurement could improve CV and mortality risk prediction, beyond traditional risk models and whether this substance may represent a therapeutic target for ameliorating clinical outcomes in this vulnerable population.

## Supplementary Information

Below is the link to the electronic supplementary material.

Supplementary file1 (DOCX 91 KB)

Supplementary file2 (DOCX 23 KB)

## Data Availability

Raw study data are available from the Corresponding Author upon reasonable request.
