# Supplementary material for: Unbalanced circulating Humanin levels and cardiovascular risk in chronic hemodialysis patients: a pilot, prospective study
Source: J Nephrol. 2024 Aug 5;37(7):1863–70. doi: 10.1007/s40620-024-02032-4 (PMC11519124; doi:10.1007/s40620-024-02032-4)

**Supplementary Figure 1.** Baseline circulating Humanin levels in HD patients reaching A) the primary composite endpoint (all-cause mortality + non-fatal CV events) and B) the secondary endpoint (mortality) during follow-up as compared to others.


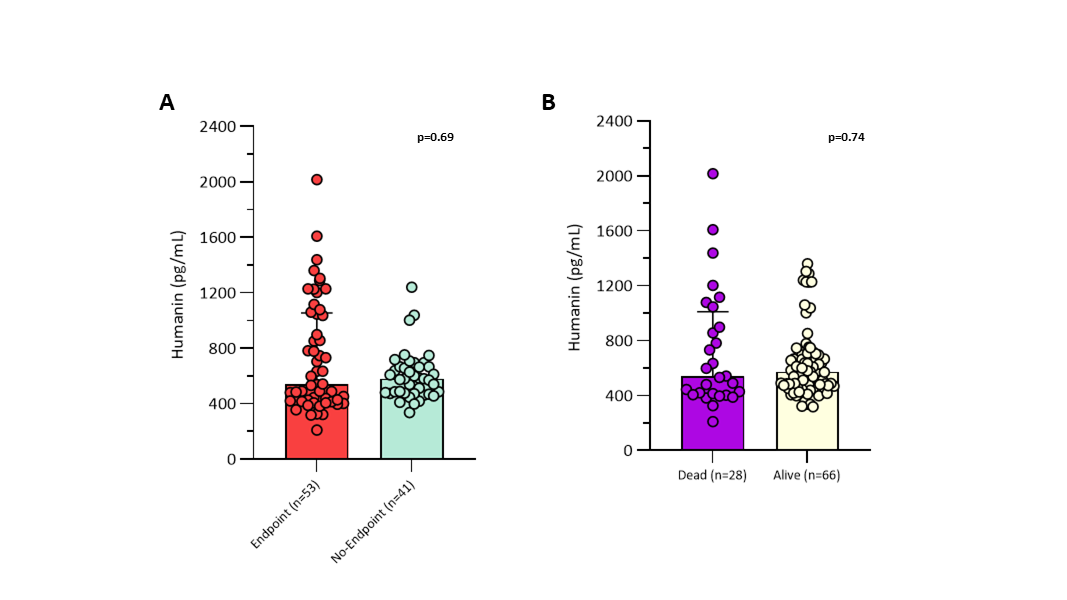

Supplement: Supplementary file 1 — Supplementary file1 (DOCX 91 KB) [file 40620_2024_2032_MOESM1_ESM.docx]
