# Supplementary material for: Unbalanced circulating Humanin levels and cardiovascular risk in chronic hemodialysis patients: a pilot, prospective study
Source: J Nephrol. 2024 Aug 5;37(7):1863–70. doi: 10.1007/s40620-024-02032-4 (PMC11519124; doi:10.1007/s40620-024-02032-4)
Supplement: Supplementary file 2 — Supplementary file2 (DOCX 23 KB) [file 40620_2024_2032_MOESM2_ESM.docx]

**Supplementary Table 1.** Main clinical characteristics of the study cohort and differences between individuals reaching the primary composite endpoint as compared to others. Statistically significant differences are highlighted in bold.

| ***Clinical parameters*** | All HD patients (n=94) | Endpoint-no  (n=41) | Endpoint-yes  (n=53) | *p for difference* |
| --- | --- | --- | --- | --- |
| **Age (yrs)** | **68±12.8** | **63.8±12.8** | **71.2±11.9** | **0.005** |
| Males n (%) | 68 (63.9) | 28 (68.3) | 40 (75.5) | 0.49 |
| Dry weight (kg) | 71.9±15.2 | 70.9±15.6 | 72.7±15.1 | 0.64 |
| BMI (kg/m^2^) | 25.7±5.6 | 24.8±4.5 | 26.4±6.2 | 0.18 |
| Waist-Hip ratio (cm) | 0.97±0.11 | 0.97±0.14 | 0.97±0.09 | 0.82 |
| SBP (mmHg) | 141.2±22.5 | 140.7±20 | 141.6±24.4 | 0.85 |
| **DBP (mmHg)** | **72.5±12.1** | **75.5±10.8** | **69.2±12.1** | **0.03** |
| **Pulse pressure (mmHg)** | **68.7±21.6** | **65.1±18.1** | **72.3±23.7** | **0.04** |
| ***Dialysis treatment*** |  |  |  |  |
| Hemodialysis n (%) | 54 (57.4) | 21(51.2) | 33 (62.3) | 0.30 |
| Hemodiafiltration n (%) | 40 (42.6) | 20(48.8) | 20 (37.7) |  |
| Kt/V | 1.42±0.27 | 1.43±0.24 | 1.45±0.29 | 0.84 |
| Dialysis vintage (mo.) | 33 [17-75.2] | 32.5 [12.2-52] | 50.5 [15.5-105] | 0.43 |
| ***Comorbidities*** |  |  |  |  |
| Ischemic Heart Disease n (%) | 31 (32.9) | 15 (36.6) | 16 (30.2) | 0.77 |
| Stroke/TIA n (%) | 11 (11.7) | 3 (7.3) | 8 (15.1) | 0.33 |
| Peripheral vasculopathy n (%) | 28 (29.8) | 11 (26.8) | 17 (37.1) | 0.45 |
| **Diabetes n (%)** | **26 (27.6)** | **5 (12.2)** | **21 (39.6)** | **0.003** |
| ***Echocardiography*** |  |  |  |  |
| **LAVi (mL/m^2^)** | **28.8 [19.9-39.8]** | **23.2 [17.5-42.9]** | **34.5 [24.7-42.7]** | **0.04** |
| LAD (cm) | 4.2±0.92 | 3.70±0.50 | 5.39±0.84 | 0.39 |
| **LVMi (g/m^2^)** | **136.8±38.8** | **124.7±38** | **141.9±40.1** | **0.03** |
| **Ejection Fraction (%)** | **57.1±8.3** | **59.6±7.6** | **55.9±9.9** | **0.05** |
| Vmax (m/sec) | 2.48±0.86 | 2.38±0.51 | 2.98±0.54 | 0.33 |
| **E/e’** | **10.8 [7.9-12.2]** | **8.4 [6.2-11.6]** | **11.9 [7.9-14.4]** | **0.03** |
| RAVi (mL/m^2^) | 21.5±8.8 | 20.1±9.2 | 22.6±10.4 | 0.36 |
| ***Laboratory values*** |  |  |  |  |
| Glycemia (mg/dL) | 102.3±12.4 | 88.5±9.94 | 114±14 | 0.27 |
| HbA1c (%) | 4.2±0.3 | 4.0±0.5 | 5.9[4.2-6.4] | 0.35 |
| Serum creatinine (mg/dL) | 9.93±3.2 | 9.50±2.1 | 10.1±2.4 | 0.47 |
| Urea (mg/dL) | 135.9±34.5 | 140.6±35.2 | 132.4±33.8 | 0.25 |
| Sodium (mg/dL) | 136.7±13.7 | 139.5±3.1 | 134.8±2.7 | 0.13 |
| Potassium (mg/dL) | 4.97±0.70 | 4.93±0.68 | 5.01±0.71 | 0.59 |
| Phosphate (mg/dL) | 4.75±0.54 | 4.76±0.41 | 4.75±0.69 | 0.98 |
| Calcium (mg/dL) | 9.17±0.65 | 9.28±1 | 8.80±0.22 | 0.15 |
| iPTH (pg/mL) | 236.6 [135.1-381.2] | 414.5 [209.5-552.3] | 257.5 [190.1-336] | 0.27 |
| Uric acid (mg/dL) | 5.81±1.13 | 5.79±0.77 | 5.82±1.78 | 0.92 |
| Albumin (g/dL) | 4.02±0.75 | 4.06±0.44 | 3.99±0.30 | 0.84 |
| ALP (U/L) | 78.5 [60-89.2] | 75.5 [39-112] | 83.5 [61-84.5] | 0.66 |
| Total Cholesterol (mg/dL) | 148±43.3 | 150.8±34.7 | 145.8±49.1 | 0.58 |
| **HDL (mg/dL)** | **41.6±10.3** | **46.3±10.1** | **40.3±10.2** | **0.03** |
| Triglycerides (mg/dL) | 127.5 [86-196.5] | 175.5 [121.2-321.2] | 127 [71.7-308] | 0.55 |
| C-reactive protein (mg/L) | 5.05 [1-3.2] | 6.3 [3.23-8.8] | 5.85 [4-7.47] | 0.96 |
| Fibrinogen (mg/dL) | 422.7±38.6 | 410±21.1 | 432±35.1 | 0.35 |
| Haemoglobin (g/dL) | 10.9±1.1 | 11±1 | 10.9±1.2 | 0.88 |
| RBC (n x10^3^) | 3.75±0.70 | 3.64±0.45 | 3.83±0.84 | 0.20 |
| WBC (n x10^3^) | 6.93±3.1 | 6.46±2.1 | 7.29±3 | 0.20 |
| PLT (n x 10^3^) | 223.1±73.8 | 231±72.8 | 216±81.5 | 0.34 |
| TSAT (%) | 22 [19.5-35.4] | 23.7 [15.2-43.2] | 21.7 [17.4-30.8] | 0.22 |
| Ferritin (mcg/L) | 303.5 [102.4-344] | 319 [151.2-447.7] | 266 [141.2-414.7] | 0.63 |
| Humanin (pg/mL) | 575 [450.7-759.5] | 543[419.2-1050.5] | 580 [476.2-667] | 0.69 |

**Legend:** ALP, Alkaline phosphatase; BMI, Body mass index; DBP, diastolic blood pressure; E/e’, early diastolic peak left ventricular inflow velocity (E)/early diastolic peak lateral mitral annular velocity (e’) ratio; ESR, erythrocyte sedimentation rate; HDL, high density lipoprotein; LAVi, left atrial volume index; LAD: left atrial diameter; LDL, low density lipoprotein; LVMi, left ventricular mass index; PLT, platelet count; iPTH, intact parathormone; RAVi, right atrial volume index; RBC, red blood cell count; SBP, systolic blood pressure; TIA: transient ischemic attack; TSAT, transferrin saturation; Vmax: Peak aortic valve velocity; WBC, white blood cell count.
